# Supplementary material for: Nairobi Sheep Disease Virus: A Historical and Epidemiological Perspective
Source: Front Vet Sci. 2020 Jul 22;7:419. doi: 10.3389/fvets.2020.00419 (PMC7387652; doi:10.3389/fvets.2020.00419)
Supplement: Supplementary file 1 [file Data_Sheet_1.PDF]

Nairobi Sheep Disease Virus: a historical and epidemiological perspective

Running title: Nairobi Sheep Disease Virus distribution

Stephanie Krasteva[1], Manuel Jara[1], Alba Frias-De-Diego[1], Gustavo Machado[1*]

[1]Department of Population Health and Pathobiology, North Carolina State University, College of Veterinary Medicine, North Carolina, United States of America.

[*] **Correspondence**
Dr. Gustavo Machado
gmachad@ncsu.edu

Table S1: Data points extracted from systematic literature review.

Author	Type	Tick Species	Country	Locality and State	Latitude	Longitude	Host
Gong et al., 2013	Tick	*H. longicornis*	China	Jian, Jilin	40.8666667	125.566667	Sheep
	Tick	*H. longicornis*	China	Jinxing, Jilin	42.4166667	130.633333	Cattle
	Tick	*H. longicornis*	China	Dadong, Liaoning	40.1166667	124.383333	Sheep
Dandawate and Shah, 1969	Tick	*H. intermedia*	India	Ganjam, Orissa	19.387388	85.051544	Goat
	Host		India	Bhanjanagar, Orissa	19.9358071	84.582481	Goat
	Host		India	Arehally, Mysore	13.0462575	75.8040022	Goat
	Host		India	Zeban, Kashmir	33.7125334	74.9866287	Sheep
Joshi et al., 2005	Tick	*H. intermedia, R. hemaphysaloides*	India	Manjari, Maharashtra	19.4822619	74.7900264	Goat
	Tick	*H. intermedia*	India	Saswad, Maharashtra	18.3463128	74.0301826	Sheep, Goat
	Tick	*H. intermedia*	India	Paud, Maharashtra	18.5243308	73.6138821	Goat
	Tick	*H. intermedia*	India	Loni, Maharashtra	19.58041	74.4659065	Goat
Boshell, 1970	Tick	*H. intermedia*	India	Hubballi, Karnataka	15.3647083	75.1239547	Sheep, Goat
	Tick	*H. intermedia*	India	Barur, Tamil Nadu	12.305112	78.305268	Sheep, Goat
	Tick	*H. intermedia*	India	Sagar, Madhya Pradesh	23.838805	78.7378068	Sheep, Goat
Joshi et al., 1998	Host		India	Veerapuram, Tamil Nadu	12.554789	80.093853	Sheep, Goat, Human
Perera et al., 1996	Tick, Host	*H. intermedia*	Sri Lanka	Kottukachchiya (Breeding Station)	7.925625	79.969421	Goat, Human
Weinbren et al., 1958	Host		Uganda	Kawanda	0.422329	32.541517	Sheep
	Host		Uganda	Kamuli	0.944785	33.126717	Goat
	Host		Uganda	Kampiringisa	0.189173	32.246467	Sheep
	Host		Uganda	Mbarara	-0.60716	30.654502	Sheep
	Host		Uganda	Entebbe	0.051184	32.463708	Goat
Jesset, 1978	Host		Tanzania	Arusha	-3.386925	36.682993	Sheep, Goat
	Host		Tanzania	Mwanza	-2.51643	32.917452	Sheep, Goat
	Host		Tanzania	Mpwapwa	-6.347795	36.48512	Sheep, Goat
	Host		Tanzania	Dar-es-Salaam	-6.792354	39.208328	Sheep, Goat

	Host	Tanzania	Iringa	-7.773094	35.69912	Sheep, Goat
	Host	Tanzania	Loliondo	-2.05	35.616667	Sheep, Goat
	Host	Tanzania	Tabora	-5.042495	32.819733	Sheep, Goat
Terpestra, 1969	Host	Uganda	Okoro, West Nile	2.5544293	30.9417368	Sheep
	Host	Uganda	Ayivu, West Nile	3.037925	30.9417368	Goat
	Host	Uganda	Maracha, West Nile	3.2873127	30.9403023	Goat
	Host	Uganda	Terego, West Nile	3.1710085	31.1252135	Goat
	Host	Uganda	Koboko, West Nile	3.4167992	30.9589496	Sheep, Goat
	Host	Uganda	Aringa, West Nile	3.4448673	31.1975236	Goat
	Host	Uganda	Bujenje, Bunyoro	1.5939148	31.5826642	Sheep, Goat
	Host	Uganda	Buruli, Bunyoro	1.3489721	32.4467238	Sheep, Goat
	Host	Uganda	Bugahya, Bunyoro	1.6343426	31.1710389	Sheep, Goat
	Host	Uganda	Mwenge, Toro	0.7137652	30.6199895	Sheep, Goat
	Host	Uganda	Burahya, Toro	0.6832535	30.2974199	Sheep, Goat
	Host	Uganda	Bunyangabu, Toro	0.4870918	30.2051096	Sheep, Goat
	Host	Uganda	Busongora, Toro	0.3244003	30.0665236	Goat
	Host	Uganda	Bujumbura, Kigezi	-0.5546336	29.8352303	Sheep, Goat
	Host	Uganda	Kinkizi, Kigezi	-0.8195253	29.742604	Goat
	Host	Uganda	Rukiga, Kigezi	-1.1326337	30.043412	Sheep, Goat
	Host	Uganda	Bufumbira, Kigezi	-1.1538569	29.6499162	Sheep, Goat
	Host	Uganda	Ndorwa, Kigezi	-1.3504705	30.0202964	Sheep, Goat
	Host	Uganda	Tororo, Bukedi	0.6782274	34.1865669	Sheep
	Host	Uganda	Samia Bugwe, Bukedi	0.4044731	34.0195827	Goat
Edelsten, 1975	Host	Somalia		10.34	43.113	Sheep, Goat
	Host	Somalia		10.286	43.443	Sheep, Goat
	Host	Somalia		10.064	43.209	Sheep, Goat
	Host	Somalia		9.907	43.513	Sheep, Goat
	Host	Somalia		9.783	43.207	Sheep, Goat
	Host	Somalia		9.741	43.773	Sheep, Goat
	Host	Somalia		9.867	44.304	Sheep, Goat
	Host	Somalia		9.476	43.504	Sheep, Goat
	Host	Somalia		9.439	43.797	Sheep, Goat
	Host	Somalia		9.203	43.935	Sheep, Goat
	Host	Somalia		9.224	44.41	Sheep, Goat
	Host	Somalia		8.969	44.391	Sheep, Goat
	Host	Somalia		9.427	44.887	Sheep, Goat
	Host	Somalia		9.9	44.782	Sheep, Goat
	Host	Somalia		9.75	45.179	Sheep, Goat
	Host	Somalia		8.833	44.824	Sheep, Goat
	Host	Somalia		8.709	45.154	Sheep, Goat
	Host	Somalia		9.481	45.348	Sheep, Goat
	Host	Somalia		9.161	45.409	Sheep, Goat
	Host	Somalia		8.489	45.776	Sheep, Goat

	Host	Somalia	8.667	46.01	Sheep, Goat
	Host	Somalia	9.041	46.347	Sheep, Goat
	Host	Somalia	8.358	46.143	Sheep, Goat
	Host	Somalia	8.693	46.513	Sheep, Goat
	Host	Somalia	9.259	46.679	Sheep, Goat
	Host	Somalia	9.554	47.126	Sheep, Goat
	Host	Somalia	8.744	47.051	Sheep, Goat
	Host	Somalia	8.281	46.679	Sheep, Goat
	Host	Somalia	8.576	47.484	Sheep, Goat
	Host	Somalia	8.077	47.119	Sheep, Goat
	Host	Somalia	8.07	47.875	Sheep, Goat
	Host	Somalia	8.487	48.085	Sheep, Goat
	Host	Somalia	9.238	48.34	Sheep, Goat
	Host	Somalia	9.862	48.614	Sheep, Goat
	Host	Somalia	9.659	49.115	Sheep, Goat
Davies, 1978[1]	Host	Kenya	1.494	35.524	Sheep, Goat
	Host	Kenya	1.384	35.517	Sheep, Goat
	Host	Kenya	1.346	35.402	Sheep, Goat
	Host	Kenya	1.247	35.27	Sheep, Goat
	Host	Kenya	1.315	35.616	Sheep, Goat
	Host	Kenya	1.205	35.699	Sheep, Goat
	Host	Kenya	1.111	35.69	Sheep, Goat
	Host	Kenya	0.885	35.569	Sheep, Goat
	Host	Kenya	0.788	35.562	Sheep, Goat
	Host	Kenya	0.758	35.43	Sheep, Goat
	Host	Kenya	0.676	35.572	Sheep, Goat
	Host	Kenya	0.581	35.574	Sheep, Goat
	Host	Kenya	0.452	35.6	Sheep, Goat
	Host	Kenya	0.376	35.442	Sheep, Goat
	Host	Kenya	0.771	35.314	Sheep, Goat
	Host	Kenya	0.606	35.257	Sheep, Goat
	Host	Kenya	0.685	34.573	Sheep, Goat
	Host	Kenya	0.716	34.755	Sheep, Goat
	Host	Kenya	0.635	34.803	Sheep, Goat
	Host	Kenya	0.043	34.341	Sheep, Goat
	Host	Kenya	0.165	34.556	Sheep, Goat
	Host	Kenya	0.19	35.009	Sheep, Goat
	Host	Kenya	0.399	35.19	Sheep, Goat
	Host	Kenya	0.336	35.165	Sheep, Goat
	Host	Kenya	0.264	35.156	Sheep, Goat
	Host	Kenya	0.218	35.193	Sheep, Goat

Host	Kenya	0.138	35.152	Sheep, Goat
Host	Kenya	0.085	35.214	Sheep, Goat
Host	Kenya	0.173	35.285	Sheep, Goat
Host	Kenya	0.426	35.782	Sheep, Goat
Host	Kenya	0.276	35.839	Sheep, Goat
Host	Kenya	-0.838	34.24	Sheep, Goat
Host	Kenya	-0.718	34.286	Sheep, Goat
Host	Kenya	-0.236	35.546	Sheep, Goat
Host	Kenya	-0.235	35.665	Sheep, Goat
Host	Kenya	-0.33	35.536	Sheep, Goat
Host	Kenya	-0.506	34.859	Sheep, Goat
Host	Kenya	-0.465	35.037	Sheep, Goat
Host	Kenya	-0.494	35.192	Sheep, Goat
Host	Kenya	-0.875	34.763	Sheep, Goat
Host	Kenya	-0.807	34.831	Sheep, Goat
Host	Kenya	-0.755	35.007	Sheep, Goat
Host	Kenya	-0.826	35.138	Sheep, Goat
Host	Kenya	-0.935	35.149	Sheep, Goat
Host	Kenya	-0.948	35.016	Sheep, Goat
Host	Kenya	-1.037	35.066	Sheep, Goat
Host	Kenya	-1.127	34.873	Sheep, Goat
Host	Kenya	-1.299	34.868	Sheep, Goat
Host	Kenya	-1.107	35.101	Sheep, Goat
Host	Kenya	-1.203	35.056	Sheep, Goat
Host	Kenya	-1.287	35.185	Sheep, Goat
Host	Kenya	-1.132	35.307	Sheep, Goat
Host	Kenya	-1.312	35.321	Sheep, Goat
Host	Kenya	-1.175	35.472	Sheep, Goat
Host	Kenya	-0.966	35.501	Sheep, Goat
Host	Kenya	-1	35.82	Sheep, Goat
Host	Kenya	-1.049	36.039	Sheep, Goat
Host	Kenya	-1.202	35.838	Sheep, Goat
Host	Kenya	-1.544	35.639	Sheep, Goat
Host	Kenya	-1.437	35.88	Sheep, Goat
Host	Kenya	-1.827	35.727	Sheep, Goat
Host	Kenya	-1.781	35.873	Sheep, Goat
Host	Kenya	-0.114	36.82	Sheep, Goat
Host	Kenya	-0.202	36.921	Sheep, Goat
Host	Kenya	-0.399	36.918	Sheep, Goat
Host	Kenya	-0.477	36.957	Sheep, Goat
Host	Kenya	-0.56	36.918	Sheep, Goat

Host	Kenya	-0.738	36.5	Sheep, Goat
Host	Kenya	-0.912	36.476	Sheep, Goat
Host	Kenya	-0.902	36.695	Sheep, Goat
Host	Kenya	-1.009	36.677	Sheep, Goat
Host	Kenya	-0.967	36.774	Sheep, Goat
Host	Kenya	-1.042	36.843	Sheep, Goat
Host	Kenya	-1.084	36.789	Sheep, Goat
Host	Kenya	-1.125	36.845	Sheep, Goat
Host	Kenya	-1.184	36.877	Sheep, Goat
Host	Kenya	-1.195	36.699	Sheep, Goat
Host	Kenya	-1.048	37.01	Sheep, Goat
Host	Kenya	-0.851	36.953	Sheep, Goat
Host	Kenya	-0.96	37.064	Sheep, Goat
Host	Kenya	-0.863	37.091	Sheep, Goat
Host	Kenya	-0.7	36.954	Sheep, Goat
Host	Kenya	-0.622	37.037	Sheep, Goat
Host	Kenya	-0.725	37.077	Sheep, Goat
Host	Kenya	-0.651	37.15	Sheep, Goat
Host	Kenya	-0.559	37.184	Sheep, Goat
Host	Kenya	-0.765	37.24	Sheep, Goat
Host	Kenya	-0.559	37.26	Sheep, Goat
Host	Kenya	-1.116	37.187	Sheep, Goat
Host	Kenya	-1.174	37.087	Sheep, Goat
Host	Kenya	-1.236	36.947	Sheep, Goat
Host	Kenya	-1.228	37.044	Sheep, Goat
Host	Kenya	-1.28	37.008	Sheep, Goat
Host	Kenya	-1.394	36.728	Sheep, Goat
Host	Kenya	-1.157	37.343	Sheep, Goat
Host	Kenya	-1.518	37.051	Sheep, Goat
Host	Kenya	-1.474	37.146	Sheep, Goat
Host	Kenya	-1.498	37.346	Sheep, Goat
Host	Kenya	-1.915	37.236	Sheep, Goat
Host	Kenya	-0.809	37.327	Sheep, Goat
Host	Kenya	-0.769	37.409	Sheep, Goat
Host	Kenya	-0.657	37.376	Sheep, Goat
Host	Kenya	-0.557	37.334	Sheep, Goat
Host	Kenya	-0.582	37.51	Sheep, Goat
Host	Kenya	-0.671	37.664	Sheep, Goat
Host	Kenya	-0.759	37.712	Sheep, Goat
Host	Kenya	-0.53	37.842	Sheep, Goat
Host	Kenya	-0.482	37.634	Sheep, Goat

Host	Kenya	-0.387	37.714	Sheep, Goat
Host	Kenya	-0.347	37.528	Sheep, Goat
Host	Kenya	-0.299	37.691	Sheep, Goat
Host	Kenya	-0.23	37.722	Sheep, Goat
Host	Kenya	-0.183	37.638	Sheep, Goat
Host	Kenya	-0.139	37.725	Sheep, Goat
Host	Kenya	-0.072	37.693	Sheep, Goat
Host	Kenya	-0.038	37.625	Sheep, Goat
Host	Kenya	-0.004	37.696	Sheep, Goat
Host	Kenya	-0.021	37.796	Sheep, Goat
Host	Kenya	0.004	37.864	Sheep, Goat
Host	Kenya	-0.323	37.958	Sheep, Goat
Host	Kenya	-0.387	38.062	Sheep, Goat
Host	Kenya	0.099	37.926	Sheep, Goat
Host	Kenya	0.127	37.825	Sheep, Goat
Host	Kenya	0.192	37.885	Sheep, Goat
Host	Kenya	0.192	37.993	Sheep, Goat
Host	Kenya	0.283	37.987	Sheep, Goat
Host	Kenya	-2.927	37.486	Sheep, Goat
Host	Kenya	-2.919	37.623	Sheep, Goat
Host	Kenya	-3.052	37.715	Sheep, Goat
Host	Kenya	-3.431	37.748	Sheep, Goat
Host	Kenya	-3.395	37.903	Sheep, Goat
Host	Kenya	-3.486	37.797	Sheep, Goat
Host	Kenya	-3.466	38.049	Sheep, Goat
Host	Kenya	-3.491	38.145	Sheep, Goat
Host	Kenya	-3.55	38.333	Sheep, Goat
Host	Kenya	-3.588	38.38	Sheep, Goat
Host	Kenya	-3.62	38.461	Sheep, Goat
Host	Kenya	-3.459	38.385	Sheep, Goat
Host	Kenya	-3.402	38.455	Sheep, Goat
Host	Kenya	-3.546	38.479	Sheep, Goat
Host	Kenya	-3.566	38.602	Sheep, Goat
Host	Kenya	-3.649	38.651	Sheep, Goat
Host	Kenya	-3.819	38.7	Sheep, Goat
Host	Kenya	-3.87	38.674	Sheep, Goat
Host	Kenya	-3.886	38.754	Sheep, Goat
Host	Kenya	-4.235	39.581	Sheep, Goat
Host	Kenya	-4.01	39.616	Sheep, Goat
Host	Kenya	-4.015	39.676	Sheep, Goat
Host	Kenya	-3.961	39.701	Sheep, Goat

	Host	Kenya	-3.891	39.735	Sheep, Goat
	Host	Kenya	-3.759	39.79	Sheep, Goat
	Host	Kenya	-3.646	39.561	Sheep, Goat
	Host	Kenya	-3.63	39.777	Sheep, Goat
	Host	Kenya	-3.588	39.852	Sheep, Goat
	Host	Kenya	-3.458	39.905	Sheep, Goat
Davies, 1978[2]	Host	Kenya	0.282	35.034	Sheep, Goat
	Host	Kenya	0.163	35.097	Sheep, Goat
	Host	Kenya	0.617	35.097	Sheep, Goat
	Host	Kenya	0.536	35.253	Sheep, Goat
	Host	Kenya	-0.096	36.74	Sheep, Goat
	Host	Kenya	-0.187	36.957	Sheep, Goat
	Host	Kenya	-0.288	36.698	Sheep, Goat
	Host	Kenya	-0.428	37.164	Sheep, Goat
	Host	Kenya	-1.147	36.355	Sheep, Goat
	Host	Kenya	-0.929	36.513	Sheep, Goat
	Host	Kenya	-0.879	36.617	Sheep, Goat
	Host	Kenya	-1.249	36.515	Sheep, Goat
	Host	Kenya	-1.36	36.55	Sheep, Goat
	Host	Kenya	-1.293	36.607	Sheep, Goat
	Host	Kenya	-1.191	36.619	Sheep, Goat
	Host	Kenya	-1.086	36.601	Sheep, Goat
	Host	Kenya	-1.13	36.713	Sheep, Goat
	Host	Kenya	-1.239	36.7	Sheep, Goat
	Host	Kenya	-1.345	36.693	Sheep, Goat
	Host	Kenya	-1.027	36.739	Sheep, Goat
	Host	Kenya	-0.977	36.829	Sheep, Goat
	Host	Kenya	-1.088	36.817	Sheep, Goat
	Host	Kenya	-1.205	36.798	Sheep, Goat
	Host	Kenya	-1.301	36.777	Sheep, Goat
	Host	Kenya	-1.161	36.881	Sheep, Goat
	Host	Kenya	-1.059	36.916	Sheep, Goat
	Host	Kenya	-0.844	36.998	Sheep, Goat
	Host	Kenya	-0.979	36.996	Sheep, Goat
	Host	Kenya	-1.11	36.993	Sheep, Goat
	Host	Kenya	-1.046	37.095	Sheep, Goat
	Host	Kenya	-1.191	37.084	Sheep, Goat
	Host	Kenya	-1.206	36.957	Sheep, Goat
	Host	Kenya	-1.28	36.88	Sheep, Goat
	Host	Kenya	-1.379	36.836	Sheep, Goat
	Host	Kenya	-1.42	36.772	Sheep, Goat

Host	Kenya	-1.359	36.938	Sheep, Goat
Host	Kenya	-1.452	36.911	Sheep, Goat
Host	Kenya	-1.442	36.992	Sheep, Goat
Host	Kenya	-1.402	37.09	Sheep, Goat
Host	Kenya	-1.312	37.134	Sheep, Goat
Host	Kenya	-1.291	37.034	Sheep, Goat
Host	Kenya	-0.969	37.24	Sheep, Goat
Host	Kenya	-0.984	37.42	Sheep, Goat
Host	Kenya	-1.647	37.185	Sheep, Goat
Host	Kenya	-1.759	37.176	Sheep, Goat
Host	Kenya	-1.775	37.297	Sheep, Goat
Host	Kenya	-2.857	37.439	Sheep, Goat
Host	Kenya	-2.886	37.578	Sheep, Goat
Host	Kenya	-2.977	37.668	Sheep, Goat
Host	Kenya	-4.271	39.529	Sheep, Goat
Host	Kenya	-4.087	39.601	Sheep, Goat
Host	Kenya	-4.034	39.483	Sheep, Goat
Host	Kenya	-4.008	39.58	Sheep, Goat
Host	Kenya	-3.988	39.673	Sheep, Goat
Host	Kenya	-3.912	39.624	Sheep, Goat
Host	Kenya	-3.915	39.727	Sheep, Goat
Host	Kenya	-3.903	39.512	Sheep, Goat
Host	Kenya	-3.873	39.411	Sheep, Goat
Host	Kenya	-3.812	39.583	Sheep, Goat
Host	Kenya	-3.827	39.671	Sheep, Goat
Host	Kenya	-3.816	39.765	Sheep, Goat
Host	Kenya	-3.7	39.573	Sheep, Goat
Host	Kenya	-3.719	39.657	Sheep, Goat
Host	Kenya	-3.78	39.474	Sheep, Goat
Host	Kenya	-3.707	39.764	Sheep, Goat
Host	Kenya	-3.615	39.642	Sheep, Goat
Host	Kenya	-3.551	39.73	Sheep, Goat
Host	Kenya	-3.609	39.821	Sheep, Goat
Host	Kenya	-3.452	39.757	Sheep, Goat
Host	Kenya	-3.469	39.877	Sheep, Goat

[1] Davies, F. G. "A survey of Nairobi sheep disease antibody in sheep and goats, wild ruminants and rodents within Kenya." Epidemiology & Infection 81.2 (1978): 251-258.

[2] Davies, F. G. "Nairobi sheep disease in Kenya. The isolation of virus from sheep and goats, ticks and possible maintenance hosts." Epidemiology &

Infection 81.2 (1978): 259-265.

Table S2: Systematic review process steps, as described by O'Connor and Sargeant, 2014.

Pre-Step: Assemble review team and develop a systematic review protocol
SK will be the main person responsible for compiling and reviewing all of the literature on NSDv. A review protocol will be prepared in accordance with O'Connor and Sargeant's methods.

Step 1: Define the review question
For reviews about disease burden (prevalence or incidence), the format uses the acronym PO: P=Population and O=Outcome (E.F.S.A., 2010).

Our research question is as follows:
What is the prevalence of Nairobi Sheep Disease in small ruminants, and is it possible to identify and anticipate vulnerable areas and populations?

Step 2: Conduct extensive search for studies
The aim of conducting an extensive search is to ensure as many relevant results as possible are included in the review. The rationale is to reduce the bias associated with the accessibility of studies based on their outcome, sometimes called retrieval bias (O'Connor and Sargeant, 2014). In animal health and welfare reviews, the inclusion of CAB Abstracts is likely to be important, as data suggest that it provides the most comprehensive coverable of animal health topics (Grindlay et al., 2012). We chose to use EBSCOhost to search for our results because it includes CAB Abstracts.

EBSCOhost (339 total results)
CAB Abstracts (125)
CAB Abstracts Archive (83)
MEDLINE (74)
Agricola (32)
Academic Search Complete (15)
CINAHL Plus with Full Text (2)
Environment Complete (2)
OpenDissertations (2)
Wildlife and Ecology Studies Worldwide (1)
MasterFILE Premier (1)
Military & Government Collection (1)

Step 3: Select relevant studies from the results of the search
Once the citations have been retrieved, it is necessary to evaluate them and identify those relevant to the review. A series of short questions are applied to the title and abstract of each citation identified (O'Connor and Sargeant, 2014). Due to the small number of publications on NSDv, we wanted to review every publication on NSDv available. For this reason, we left our questions short and simple:
Does the title or abstract mention Nairobi Sheep Disease or Ganjam Virus?

Step 4: Collecting data from relevant studies

In this step, the results of the relevant studies are extracted (O'Connor and Sargeant, 2014). We are interested in capturing disease distribution in order to build upon our ecological model, so we collected geographic locations of NSDv occurrences.
**Step 5: Assess the risk of bias in relevant studies**<br>It is important to report the potential for bias to the end user in order to alert them to potential concerns and uncertainties about the evidence summary. Since we will be including different study designs in our review, this information will be reported to the reader (O'Connor and Sargeant, 2014).
**Step 6: Synthesize the results**<br>Frequently in animal health, animal welfare, and food safety, it is not possible to combine the results in a meta-analysis. This often occurs due to a low number of relevant studies or because of poor reporting or differences in metrics used to measure the outcomes. Therefore, the presentation of results and discussion takes the place of this step (O'Connor and Sargeant, 2014).
**Step 7: Present the results**<br>The presentation of the results of the review should include the following components:<br>- The results of the search and study selection;<br>- Summary information about the characteristics of the studies identified as relevant to the review (including those that could not be included in a meta-analysis);<br>- Risk of bias assessment for the individual studies;<br>- Outcomes reported by the relevant studies;<br>- Risk of bias across the studies.<br><br>The PRISMA Statement should be strictly adhered to, and there should be a way to present a summary of the overall assessment of the body of work, such as an evidence profile. An evidence profile provides a structured means of summarizing the risk of bias for each outcome included in the systematic review. The issues considered include inconsistency, the risk of bias in the studies, indirectness, imprecision, and other considerations (O'Connor and Sargeant, 2014).
**Step 8: Interpret the results and discussion**<br>Conclusions about the results of the review and a discussion of potential biases of the review should be discussed. PRISMA guidelines recommend the discussion and interpretation include a summary of the evidence, a discussion of the limitations of the review, and the overall conclusions (O'Connor and Sargeant, 2014).

Table S3: PRISMA checklist (Moher et al., 2009).

Section/topic	#	Checklist item	Reported on page #
**TITLE**			
Title	1	Identify the report as a systematic review, meta-analysis, or both.	
**ABSTRACT**			
Structured summary	2	Provide a structured summary including, as applicable: background; objectives; data sources; study eligibility criteria, participants, and interventions; study appraisal and synthesis methods; results; limitations; conclusions and implications of key findings; systematic review registration number.	1,2
**INTRODUCTION**			
Rationale	3	Describe the rationale for the review in the context of what is already known.	8
Objectives	4	Provide an explicit statement of questions being addressed with reference to participants, interventions, comparisons, outcomes, and study design (PICOS).	Table S2
**METHODS**			
Protocol and registration	5	Indicate if a review protocol exists, if and where it can be accessed (e.g., Web address), and, if available, provide registration information including registration number.	NA
Eligibility criteria	6	Specify study characteristics (e.g., PICOS, length of follow-up) and report characteristics (e.g., years considered, language, publication status) used as criteria for eligibility, giving rationale.	Figure 2, Table S2
Information sources	7	Describe all information sources (e.g., databases with dates of coverage, contact with study authors to identify additional studies) in the search and date last searched.	9
Search	8	Present full electronic search strategy for at least one database, including any limits used, such that it could be repeated.	8
Study selection	9	State the process for selecting studies (i.e., screening, eligibility, included in systematic review, and, if applicable, included in the meta-analysis).	9
Data collection process	10	Describe method of data extraction from reports (e.g., piloted forms, independently, in duplicate) and any processes for obtaining and confirming data from investigators.	9
Data items	11	List and define all variables for which data were sought (e.g., PICOS, funding sources) and any assumptions and simplifications made.	NA
Risk of bias in individual studies	12	Describe methods used for assessing risk of bias of individual studies (including specification of whether this was done at the study or outcome level), and how this information is to be used in any data synthesis.	NA
Summary measures	13	State the principal summary measures (e.g., risk ratio, difference in means).	NA
Synthesis of results	14	Describe the methods of handling data and combining results of studies, if done, including measures of consistency (e.g., I^2) for each meta-analysis.	NA
Risk of bias across studies	15	Specify any assessment of risk of bias that may affect the cumulative evidence (e.g., publication bias, selective reporting within studies).	NA
Additional analyses	16	Describe methods of additional analyses (e.g., sensitivity or subgroup analyses, meta-regression), if done, indicating which were pre-specified.	NA
**RESULTS**			

Study selection	17	Give numbers of studies screened, assessed for eligibility, and included in the review, with reasons for exclusions at each stage, ideally with a flow diagram.	Figure 2
Study characteristics	18	For each study, present characteristics for which data were extracted (e.g., study size, PICOS, follow-up period) and provide the citations.	Table S1
Risk of bias within studies	19	Present data on risk of bias of each study and, if available, any outcome level assessment (see item 12).	NA
Results of individual studies	20	For all outcomes considered (benefits or harms), present, for each study: (a) simple summary data for each intervention group (b) effect estimates and confidence intervals, ideally with a forest plot.	NA
Synthesis of results	21	Present the main results of the review. If meta-analyses are done, include for each, confidence intervals and measures of consistency" in accordance with the text in the Explanation and Elaboration document.	13
Risk of bias across studies	22	Present results of any assessment of risk of bias across studies (see Item 15).	NA
Additional analysis	23	Give results of additional analyses, if done (e.g., sensitivity or subgroup analyses, meta-regression [see Item 16]).	NA
**DISCUSSION**			
Summary of evidence	24	Summarize the main findings including the strength of evidence for each main outcome; consider their relevance to key groups (e.g., healthcare providers, users, and policy makers).	17
Limitations	25	Discuss limitations at study and outcome level (e.g., risk of bias), and at review-level (e.g., incomplete retrieval of identified research, reporting bias).	19
Conclusions	26	Provide a general interpretation of the results in the context of other evidence, and implications for future research.	20
**FUNDING**			
Funding	27	Describe sources of funding for the systematic review and other support (e.g., supply of data); role of funders for the systematic review.	21
